# Progress in Surgery

**Published:** 1896-08-15

**Authors:** 


### Progress in Surgery.

### SURGERY OF THE ŒSOPHAGUS AND STOMACH.

**Foreign Bodies in the Œsophagus** cannot be located, says Silver,[1] with the ordinary sponge probing. The best instrument for such exploration is a *bougie à boule*, provided with several sizes of bulbs and a graduated metal stem. It should be remembered in exploring the œsophagus that there are two natural constrictions —one at the cricoid cartilage, or seven and a half inches from the teeth; the other at the cardiac orifice, or fourteen and a half inches from the teeth. Where the object to be reached is distant thirteen inches or more from the teeth gastrotomy is the proper operation; if less than thirteen inches œsophagotomy. Suturing the œsophageal wound is not essential, though desirable when the tissues are healthy. The external wound should never be completely closed, and no food should be administered by the mouth for the first twenty-four hours after operation. Morton[2] records an unsuccessful case of gastrotomy for removal of a denture, which had been swallowed nearly two years before and had become impacted in the lower portion of the œsophagus.

**Stricture of the Œsophagus** was treated by Roberts[3] in one case by opening the œsophagus above and below the stricture and then endeavouring to dilate the constriction. The child died about a fortnight later with mediastinal suppuration. Andrews[4] treated a case by gastrotomy, with dilatation of the œsophagus from below. The patient recovered, but a subsequent gastrostomy became necessary.

**Pyloroplasty** for stenosis of the pylorus and dilatation of the stomach following gastric ulceration, continues to be attended with successful results. Paci[5] records a case accompanied by intractable vomiting, which was completely cured by the ordinary longitudinal incision and transverse stitching; and Parker[6] reports a somewhat similar condition successfully treated in much the same, but a bone bobbin was inserted so as to expedite the suturing after the method of Mayo Robson.[7]

**Pylorectomy**, according to Ferguson,[8] has been performed thirteen times in America, with eight deaths and five recoveries. He strongly favours the operation for carcinoma, for whereas under medical treatment all patients die in twelve to eighteen months, pylorectomy promises a possible cure. In 19 per cent. of over nine hundred cases of cancer of the pylorus, no adhesions whatever had been found after death, starvation having taken place before the carcinoma had reached the peritoneum, so that there would be good prospects of curing most of these. The author, after opening the abdomen, would thus perform pylorectomy: (1) Liberate the duodenum from the pylorus, unite its distal cut end to the posterior surface of the stomach with Murphy's button, and invert the proximal cut end towards the pylorus and close with sutures. The advantage of completing the gastro-duodenostomy first, is that the operation can be safely stopped at this stage, and the removal of the pylorus left for a second operation if necessary. (2) Separate the stomach from the pylorus, and close it rapidly with sutures. Should the patient now present alarming symptoms, the surgeon should again cease operating and leave the pylorus in situ in the meantime. It would be necessary to fasten it in the abdominal wound and drain it externally, which, however, would only facilitate its extirpation at another time. (3) Remove the cancerous pylorus. Spend no time in trying to use interlocking ligatures, but apply forceps after forceps, and cut the mass away. This done, the ligatures can be applied more expeditiously. Ferguson believes that shock is greatly prevented if the patient is placed upon a hot-water bed during operation. Adams[9] records a successful case of pylorectomy for carcinoma in a woman aged fifty-six. A continuous catgut stitch was used, the mucous membrane being left unstitched.

**Gastro-enterostomy.**—Walker[10] refers to the small amount of cicatricial contraction found at death in cases operated upon him two years previously. His method of suturing in these cases was as follows:—The peritoneal and mucous edges of the opening in the stomach were stitched together with the idea of preventing contraction. The jejunum was opened and the edges were treated in the same way. Senn's plates were introduced; but, before drawing the silk tight, the peritoneum of the stomach and that of the jejunum near the posterior edges of the plates were united with silk, thus forming a semi-circle of stitches guarding the opening posteriorly; the plates were then drawn tightly together, and an anterior semi-circle of stitches was inserted, thus completing the circle of guarding stitches. Mayo Robson[11] reports a successful case of gastro-enterostomy, in which he used his bone bobbin; and Eiselberg[12] believes it would be wiser to substitute jejunostomy for this operation in case of severe hæmorrhage, or where there is danger of perforation of an inoperable pyloric tumour, because, after a gastro-enterostomy, the food is liable to produce the same conditions.

**Gastrostomy** forms the subject of papers by Le Moyne Hupp[13] and Morton.[14] Both authors record cases operated upon by the method proposed independently by Ssabanejew, of Odessa, and Frank, of Vienna. Both authors speak most highly of this operation, and state that it is not followed by leakage. The chief steps of the operation are as follows: A three-inch incision is made one finger's breadth beneath the left costal margin, the muscles bluntly separated, and the peritoneum opened in the same direction. By means of a piece of silk acting as a sling a cone of stomach is drawn out for one and a-half to two inches, and the peritoneum at the base of this cone is stitched to the peritoneal edges of the incision. A second incision, half an inch long, is now made through the skin only, one inch above the costal border, parallel to and about the centre of the first. The bridge of skin between the two wounds is raised by blunt dissection, and the apex of the cone of stomach drawn underneath until it comes out at the upper opening. The original abdominal wound is then closed. The projecting portion of stomach is incised and its mucous membrane sutured to the skin margin of the smaller wound. Feeding may be immediate. Lindner[15] speaks favourably of this method and quotes several cases.

**Perforating Gastric Ulcer.**—Weir and Foote[16] give an analysis of seventy-eight cases of gastric ulcer treated by laparotomy, in twenty-three of which the operation proved successful. Mortality increases with the time which is allowed to elapse between the occurrence of perforation and operation. In doubtful cases the patient should be kept quiet in a horizontal position and nothing given by the mouth. Lavage is dangerous lest rupture of a weakened stomach and extravasation occur. After incision in the median line, the liver should be lifted and the stomach gently drawn downwards and to either side. Gentle squeezing of the organ will often expose the perforation by the escape of gas or fluid. If nothing is seen on the anterior surface the lesser omentum may be torn through at a thin place near the stomach and the posterior wall explored. If any recent adhesions exist about the perforation it is well to separate them after protecting the surrounding parts by gauze or sponges. Excision of the ulcerated portion is unnecessary, and irrigation of the peritoneum is not desirable unless the amount of fluid found during the operation is large. Drainage posteriorly on both sides and in the pelvis should then be resorted to. Blume[17] arranges the cases topographically into four groups: (1) Perforations in the region of the pylorus and duodenum; (2) Perforations in the posterior wall; (3) Perforations in the anterior wall; (4) Perforations near the cardiac orifice. In group 1 the perforation is readily found; in group 2 access to the perforation is gained by cutting through the ligamentum gastrocolicum, when the stomach may be turned upward; in group 3 the perforation is most easily found, but the acuteness of the angle formed by the costal arches may encroach seriously upon the room necessary for suturing the opening; in group 4 the perforation is situated high up behind the chest wall, and, inasmuch as the stomach cannot be pulled down, the closure of the opening is very difficult, and may have to be abandoned. The author believes that operative treatment holds out more definite hopes for recovery than medical treatment, though many cases treated "expectantly" have recovered. Barker[18] considers it very important to make sure that the space between the liver and the diaphragm is perfectly clean, and thinks that the neglect of this precaution is the cause of death in many cases that are at first relieved by operation. The hand should be slipped into the abdomen between the liver and the diaphragm, and both surfaces of the space should be repeatedly wiped with a sponge, thus bringing away any particles of food and lymph collected in it. Wiping thus with a sponge is, he believes, more efficacious than elaborate flushing. Paul[19] thinks that the published cases show that the causes of failure after operation are those originally pointed out by Gould, viz.: (1) Collapse; (2) imperfect cleansing of the peritoneum; (3) further leakage of stomach contents. When the orifice can only be imperfectly closed, it may prove wise to drain the stomach by a temporary gastrostomy. When it is quite inaccessible, it might possibly be sutured through an incision in the anterior wall of the stomach. It is not every ulcer that could be got at, even in this way. A ready way of meeting the difficulty was suggested by Maurice, who introduced an indiarubber tube into the stomach through the perforation and packed it round with gauze. The author holds that many cases of gastric ulcer can be traced to the use of corsets. Weir[20] says we must distinguish clearly between acute and sub-acute cases, and chronic ones, lasting from one to three weeks, and associated with "food abscesses." These are often confounded with pyopneumothorax. The question as to the advisability of attempting to close the opening in chronic cases is a difficult one to answer, and the decision must rest largely upon the accessibility of the perforation. Lilienthal[21] says that in making the differential diagnosis between empyama and these sub-phrenic abscesses, it should be remembered that in cases of non-sacculated empyama, on inverting the patient, the area of dulness shifts to the upper part of the thorax. In addition to the above, cases are recorded by Michaux,[22] Routier,[23] Caird,[24] Page,[25] Pasteur and Morris,[26] Kirkpatrick,[27] Dalziel,[28] and others, most of them being successful.

**Results of Operations upon the Stomach.**—Haberkant[29] has made a most exhaustive study of this subject. The statistics of mortality of various surgeons for pylorectomy vary between 35 per cent. (Czermy) and 66 6 per cent. (Lowenstein). The author collected 388 cases of gastro-enterostomy: 241 for cancer, with 136 recoveries and 105 deaths; and 47 cases for ulcer, with 35 recoveries and 12 deaths. A study of the final results is of great interest and importance. Thirty cases died one to six months after operation; twelve from six to twenty-two months after. Two cases were alive more than ten months; one, one year and eleven months; and one, two years. Of fifty-one cases that recovered from pylorectomy for carcinoma, thirteen cases were alive and well at time of report, more than one year after operation; nine of these had gone beyond two years, four beyond three years, one had gone five years and four months, and one eight years.

[1] New York Med. Journ., Dec. 21, 1895. [2] Annals of Surg., April, 1896. [3] New York Med. Journ., Nov. 16, 1895. [4] Internat. Clinic., vol. iii., series iv., 1896. [5] Am. Journ. of Med. Sc., Mar., 1896. [6] Brit. Med. Journ., Dec. 14, 1895. [7] Ibidem., July 20, 1895. [8] New York Med. Journ., April 18, 1896. [9] Brit. Med. Journ., April 18, 1896; and Glasg. Med. Journ., Feb., 1896. [10] Lancet, April 25, 1896. [11] Internat. Clinics, vol. iv., series 2, 1896. [12] Am. Med. Surgic. Bullet., Feb. 22, 1896. [13] New York Med. Journ., March 28, 1896. [14] Med. Chron., March 1896, and Med. News, Jan. 25, 1896. [15] Internat. Med. Mag. Feb. 1896, and Berlin Klin. Woch., No. 80, 1895. [16] Brit. Med. Journ., May 30, 1896, and Med. News, April 25, 1896. [17] Therapeut. Gazette, May 15, 1896. [18] Clin. Journ., April 8, 1896. [19] Therapeut. Gaz., Feb 15, 1896, and Med. Chi. Journ., No. 29, 1895. [20] Med. Week, Jan. 31, 1896. [21] Ibidem. [22] Med. Week, Mar. 20, 1896. [23] Ibidem. [24] Edin. Med. Journ., June, 1896. [25] Lancet, May 23, 1896. [26] Ibidem, Dec 21, 1896. [27] Annals of Surg., Jan., 1896. [28] Glasgow Med Journ., April, 1896. [29] Am. Med. Surg. Bullet., April 11, 1896, and Archiv. f. Klin. Chir., 1895, LI., Nos. 3 and 4.

